# Long non-coding RNA MALAT1 sponges miR-30c to promote the calcification of human vascular smooth muscle cells by regulating Runx2

**DOI:** 10.1080/0886022X.2023.2204953

**Published:** 2023-04-26

**Authors:** Ying Gong, Qing Zhong, Yunfeng Xia, Yang Wen, Hua Gan

**Affiliations:** The First Affiliated Hospital of Chongqing Medical University, Chongqing, China

**Keywords:** Vascular calcification, MALAT1, miR-30c, vascular smooth muscle cells

## Abstract

**Objectives:**

Recent evidence suggested that long non-coding RNAs (lncRNAs) and microRNAs (miRNAs) play critical roles in the pathogenesis of vascular calcification (VC). In this study, we tried to explore the expression and role of a lncRNA, i.e., metastasis-associated lung adenocarcinoma transcript 1 (MALAT1), and a miRNA, i.e., miR-30c, in VC.

**Methods:**

*In vitro* VC model was induced in human vascular smooth muscle cells (VSMCs) after 10 days culture in calcifying medium containing 2 mM Na_2_HPO_4_. Alizarin red S staining, calcium assay and western blot analysis of runt-related transcription factor 2 (Runx2) and alpha smooth muscle actin (α-SMA) were performed to evaluate VC. Knockdown of MALAT1 and up-regulation of MALAT1, miR-30c and Runx2 was performed to determine the impact of these molecules on VSMCs calcification. Dual-luciferase report assay was performed to confirm the relationship between MALAT1 and miR-30c or miR-30c and Runx2. In addition, quantitative reverse transcription PCR and western blot were used to determine gene and protein expression.

**Results:**

MALAT1 was increased, while miR-30c was decreased in calcified VSMCs. Knockdown of MALAT1 suppressed VSMCs calcification; on the contrary, up-regulation of MALAT1 promoted VSMCs calcification. The effect of MALAT1 over-expression on VSMCs calcification was reversed by upregulation of miR-30c, which was reversed again by upregulation of Runx2. Dual-luciferase report assay confirmed that there is a direct interaction between MALAT1 and miR-30c, and Runx2 is a direct target of miR-30c.

**Conclusion:**

MALAT1 over-expression promoted VSMCs calcification, which was at least partially through regulating the miR-30c/Runx2 axis.

## Introduction

Vascular calcification (VC) is an active, highly complex biological process that calcium, phosphorus and other minerals abnormally deposited in the vessel wall. Depending on where the minerals were deposited, two types of VC, i.e., intimal calcification and medial calcification, are commonly observed. Intimal calcification is linked to atherosclerosis due to accumulation of lipid and cholesterol in the intimal layer of vessel, which can cause plaque rupture and acute vessel occlusion. On the other hand, medial calcification occurs in the smooth muscle cells of the vessel medial layer and frequently presents in chronic diseases such as chronic kidney disease (CKD), diabetes mellitus (DM), cardiovascular diseases (CVDs) and osteoporosis, which induces arterial stiffness and contributes to cardiovascular morbidity and mortality [1,2]. Up to now, although various mechanisms such as inflammation, oxidative stress, mitochondrial dysfunction and autophagy, have been found to be relevant with VC, the pathological basis underlying VC is far from clear [3].

Long non-coding RNAs (lncRNAs) are a group of RNA molecules that longer than 200 nucleotides. Recent studies have suggested that lncRNAs play critical roles in VC [4]. For instance, Jeong et al. found that in a rat vascular smooth muscle cells (VSMCs) calcification model induced by inorganic phosphate, hundreds of lncRNAs were differentially expressed; among them, Lrrc75a-as1 negatively regulated VC and may serve as a therapeutic target in VC [5]. Zhang et al. demonstrated that anti-differentiation noncoding RNA (ANCR) can inhibit the osteoblastic differentiation of VSMCs and attenuate mice arterial calcification through activating autophagy [6]. Other lncRNAs such as growth arrest-specific transcript 5 (GAS5) [7], small nucleolar RNA host gene 29 (SNHG29) [8], HOX antisense intergenic RNA (HOTAIR) [9], H19 [10], taurine-upregulated gene 1 (TUG1) [11] and ES3 [12], also have been reported to be associated with VC. Metastasis-associated lung adenocarcinoma transcript 1 (MALAT1) is a highly conserved nuclear localized lncRNA in mammals. In a study performed by Xiao et al. authors found that MALAT1 was highly expressed in calcified aortic valves tissues, as well as in osteoblast-like human aortic valve interstitial cells (VICs); mechanically, MALAT1 promoted osteogenic differentiation of VICs by regulating the expression of Smad4 through sponging miR-204 [13]. By searching LncRNA Symbol with MALAT1 in the LncRNADisease v2.0 database (http://www.rnanut.net/lncrnadisease/index.php/home/search), we noticed that, except for cancers, MALAT1 also highly correlated with several VC-related diseases such as liver cirrhosis (Score = 0.9933), calcific aortic valve disease (Score = 0.9820), diabetic nephropathies (DN) (Score = 0.9630) and DM (Score = 0.9053); therefore, we speculate that MALAT1 may has a role in the pathogenesis of VC.

MicroRNAs (miRNAs) are another widely studied group of non-coding RNAs that participate in the progression of VC. Many miRNAs have been demonstrated to have roles in promoting (such as miR-221, miR-222, miR-223, miR-712, miR-714, miR-762) or protecting against VC (such as miR-30b, miR-30c, miR-125b and miR-133a) [14]. miR-30c, one of the members of the miR-30 family, has been proved to be a target of MALAT1 [15–17]; however, the role of the MALAT1/miR-30c axis in VC has never been reported.

In the present study, for the first time, we found that MALAT1 and miR-30c were differentially expressed in calcified VSMCs; mechanically, MALAT1 promoted the calcification of VSMCs, which was at least partially through regulating the miR-30c/Runx2 axis.

## Materials and methods

### Cell culture

Human aortic VSMCs (BNCC340087), purchased from Bnbio (Beijing, China), were cultured in Dulbecco’s Modified Eagle Medium (DMEM) (supplemented with 10% fetal bovine serum and 1% penicillin-streptomycin) or in calcifying medium (DMEM medium supplemented with 2 mM Na_2_HPO_4_) at 37 °C and 5% CO_2_.

### Alizarin red S staining and quantification of cell calcium content

Ten days after culture, Alizarin red S staining and quantification of cell calcium content were performed. For Alizarin red S staining, cultured VSMCs were fixed with 4% paraformaldehyde solution for 20 min at room temperature, washed with PBS three times, and stained with 1% Alizarin Red S solution (pH = 4.2) for 30 min at 37 °C. After staining, samples were washed with PBS and then observed under an inverted microscope. To quantify Ca^2+^ content of the cultured VSMCs, ultrasonic decomposition was performed after adding deionized water; next, supernatant was collected after centrifugation. Detection reagents from a commercial calcium assay kit (Nanjing Jiancheng Biotechnology Institute, Nanjing, China) were then added to supernatant following the manufacturer’s instructions. The OD value (detected at 610 nm) and protein concentration of samples measured using a BCA kit (Wanleibio, Shenyang, China) were used to determine the cell calcium content.

### Cell transfection

To explore the role of MALAT1, hsa-miR-30c-5p (miR-30c) and Runx2 in vascular calcification, lentiviral short hairpin RNA (shRNA) and expression plasmids of MALAT1 (Western Biomedical Technology Company, Chongqing, China), miR-30c mimics (Invitrogen, USA) and Runx2 over-expression vector (GenePharma Company, Shanghai, China) were transfected to VSMCs using the Lipofectamine 2000 reagent according to the manufacturer’s instructions (Life Technologies Corporation, USA). After transfection, cells were cultured in calcifying medium for another 10 days to perform all the following experiments.

### Dual-luciferase report assay

To confirm the relationship between MALAT1 and miR-30c or miR-30c and Runx2, wild-type and mutant reporter plasmids of MALAT1 or Runx2 were synthesized by GenePharma (Shanghai, China). When VSMCs had grown to 70–80% confluence, synthesized reporter plasmids were co-transfected with miR-30c mimics or negative control mimics (NC mimics) into cells by the Lipofectamine 2000 reagent according to the manufacturer’s instructions (Life Technologies Corporation, USA). 48 h after transfection, the fluorescence signal was measured using the Dual Luciferase Reporter Gene Assay Kit (Beyotime, Shanghai, China).

### Quantitative reverse transcription PCR (qRT-PCR)

Total RNA of VSMCs was extracted using RNAiso Plus (Takara, Tokyo, Japan) and reverse-transcribed to cDNA using Hifair® II 1st Strand cDNA Synthesis Kit (Yeasen, Co., Ltd, Shanghai, China) or miRNA reverse transcription PCR kit (RiboBio, China). qRT-PCR was then performed using the Hieff UNICON® Universal Blue qPCR SYBR Green Master Mix (Yeasen, Co., Ltd, Shanghai, China) on an ABI Step One Plus™ real-time PCR system (ABI Applied Biosystem, USA). Relative expression of genes was calculated using the 2^−ΔΔCt^ method. The primer sequences used in this study are shown in Table 1.

**Table 1.** The primer sequences used in this study.

Name		Primer sequences
MALAT1	Forward primer	GCATTTTGGGATGGTCTTAA
	Reverse primer	CAGCGGTACACTCCTTCTCT
Runx2	Forward primer	TCTACTATGGCACTTCGTCAGGA
	Reverse primer	ATCAGCGTCAACACCATCATTC
miR-30c	Forward primer	GCGCGTGTAAACATCCTACACT
	Reverse primer	AGTGCAGGGTCCGAGGTATT
GAPDH	Forward primer	GGGAAGGTGAAGGTCGGAGT
	Reverse primer	GGGGTCATTGATGGCAACA
U6	Forward primer	CGCAAGGATGACACGCAAAT
	Reverse primer	AAAATATGGAACGCTTCACGAAT

MALAT1: metastasis-associated lung adenocarcinoma transcript 1; Runx2: runt-related transcription factor 2; GAPDH: glyceraldehyde-3-phosphate dehydrogenase.

### Western blot

Protein of VSMCs was extracted with cold RIPA buffer (Beyotime, Shanghai, China) and measured using a BCA kit (Wanleibio, Shenyang, China). Protein lysates were then separated with SDS-PAGE and transferred to PVDF membranes. After blocked with 5% skimmed milk, membranes were incubated with primary antibodies against runt-related transcription factor 2 (Runx2) (1:1000; CST, USA), alpha smooth muscle actin (α-SMA) (1:2000; Huabio, China) and β-actin (1:2000; Huabio, China) at 4 °C overnight. After washed with TBST buffer, membranes were incubated with HRP-conjugated IgG secondary antibody (1:1000; Beyotime, China) for 1 h at room temperature. The Pierce ECL Western Blotting Substrate (Thermo, USA) was used for visualization. Quantization of bands was performed using the Image J software.

### Statistical analysis

Continuous variables are expressed as mean ± standard deviation and compared with Student’s *t* test or one-way analysis of variance using the SPSS 20.0 software (SPSS, Inc., Chicago, USA), as appropriate. *p* < 0.05 was considered significant.

## Results

### MALAT1 and miR-30c were differentially expressed in the process of VSMCs calcification

**Figure 1.**
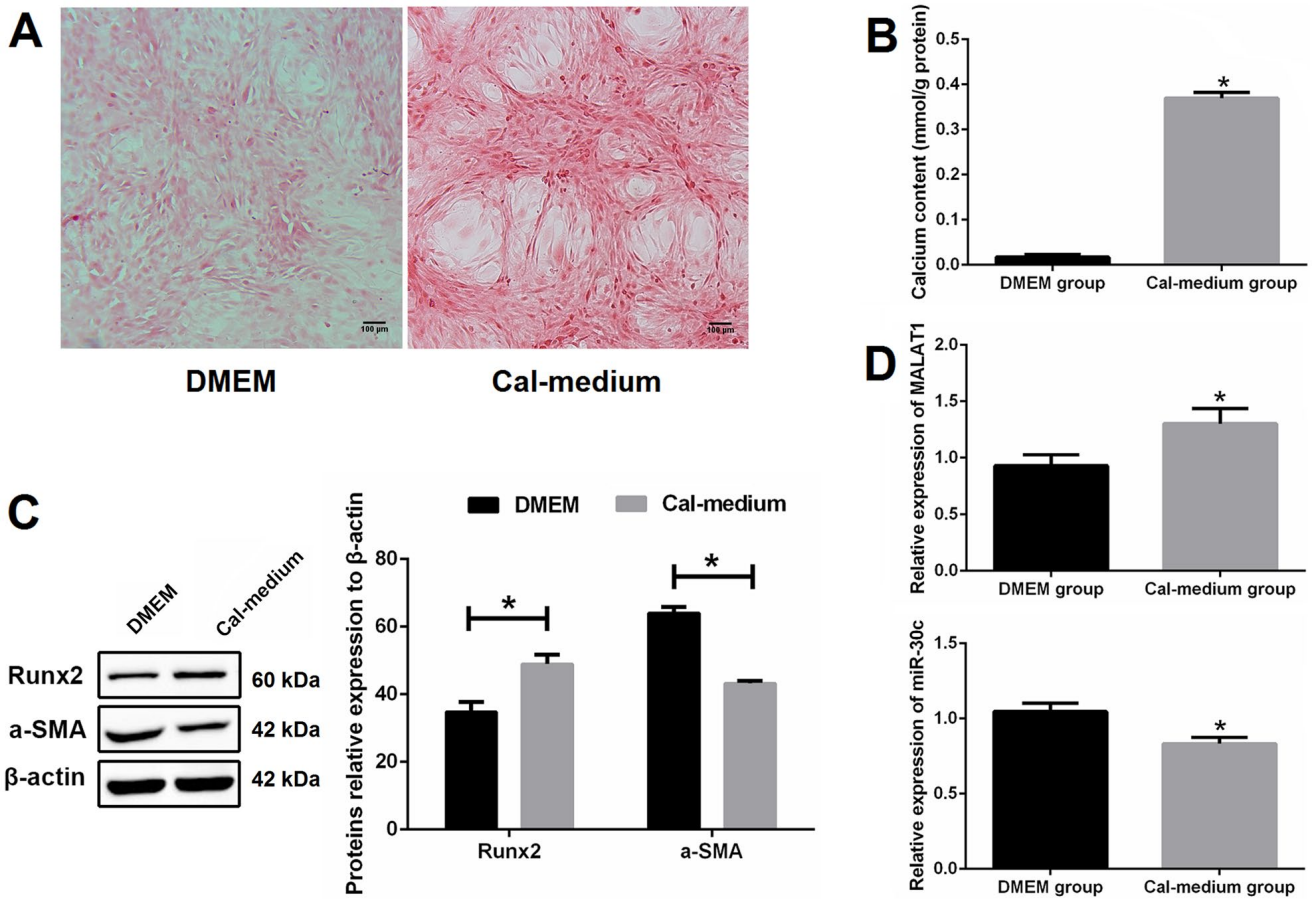
MALAT1 and miR-30c were differentially expressed in calcified VSMCs. (A) Alizarin red S staining indicated more mineralized nodules in the Cal-medium group than the DMEM group; (B) calcium assay showed higher calcium content in the Cal-medium group; (C) Western blot revealed higher expression of Runx2 and lower expression of α-SMA in the Cal-medium group; (D) qRT-PCR results showed that MALAT1 was increased and miR-30c was decreased in the Cal-medium group. MALAT1: metastasis-associated lung adenocarcinoma transcript 1; VSMCs: vascular smooth muscle cells; Cal-medium: calcifying medium; DMEM: Dulbecco’s Modified Eagle Medium; Runx2: runt-related transcription factor 2; α-SMA: alpha smooth muscle actin; qRT-PCR: quantitative reverse transcription PCR. *N* = 3. At least two independent experiments were performed. *p < 0.05 vs. the DMEM group.

To find out whether MALAT1 and miR-30c were related to VSMCs calcification, we first treated VSMCs with calcifying medium to induce *in vitro* calcification. Alizarin red S staining results showed that, after 10 days culture, more mineralized nodules were observed in the calcifying medium (Cal-medium) group as compared with the DMEM group (Figures 1(A) and S1). In line with this observation, we also found that there was higher calcium content in the Cal-medium group than the DMEM group (Figure 1(B)). Western blot results revealed that the protein expression of Runx2 was increased while α-SMA was decreased in the Cal-medium group as compared with the DMEM group (Figure 1(C)).

To verify the expression changes of MALAT1 and miR-30c in the process of VSMCs calcification, qRT-PCR was performed. Our results showed that the expression of MALAT1 was prominently increased and miR-30c was significantly decreased in the Cal-medium group as compared with the DMEM group (Figure 1(D)).

### MALAT1 promoted VSMCs calcification

**Figure 2.**
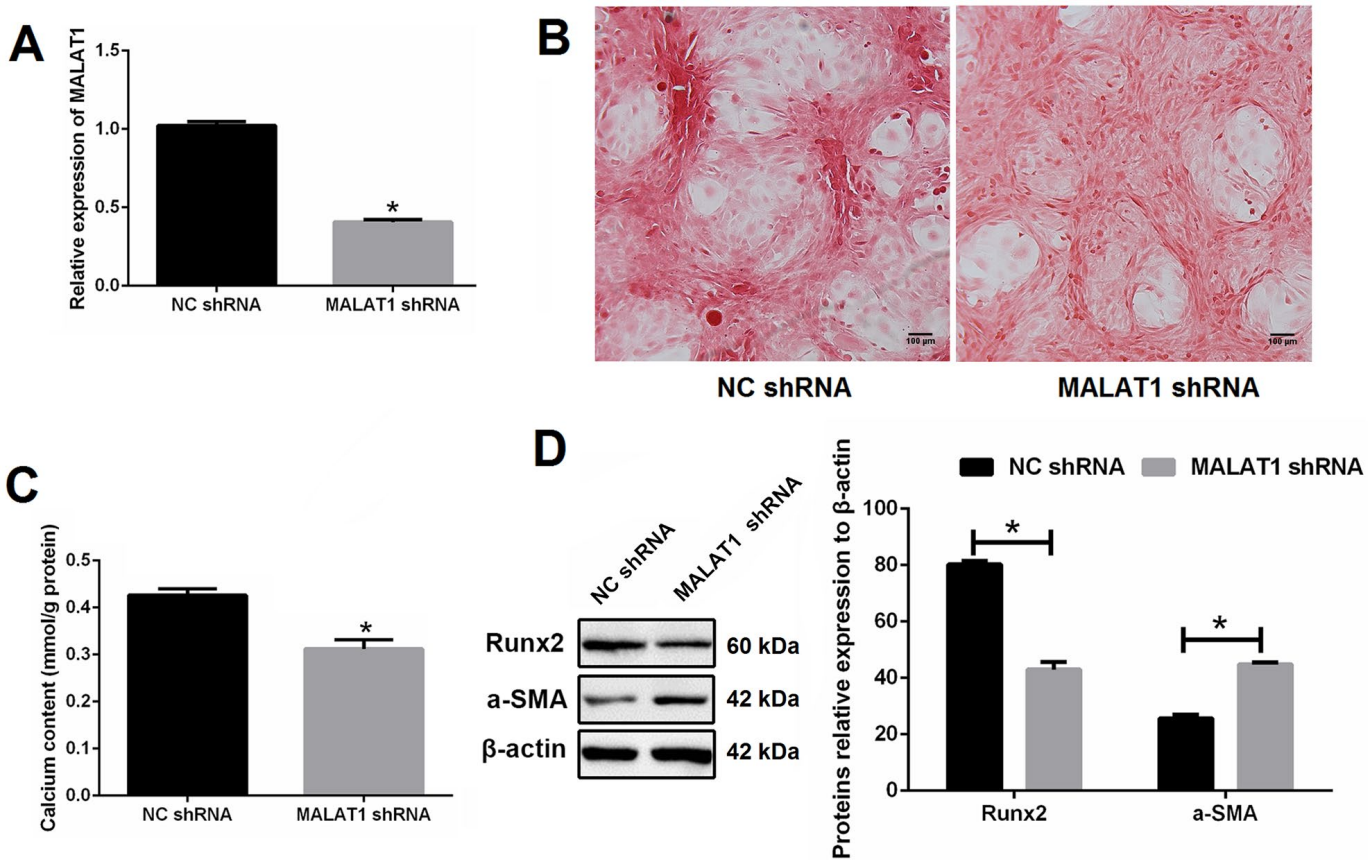
Knockdown of MALAT1 greatly suppressed VSMCs calcification. (A) qRT-PCR showed that the expression of MALAT1 was significantly decreased after MALAT1 shRNA transfection; (B) MALAT1 shRNA transfection reduced mineralized nodules as indicated by Alizarin red S staining; (C) MALAT1 shRNA transfection reduced cell calcium content as indicated by calcium assay; (D) Western blot demonstrated lower expression of Runx2 and higher expression of α-SMA after MALAT1 shRNA transfection. MALAT1: metastasis-associated lung adenocarcinoma transcript 1; VSMCs: vascular smooth muscle cells; qRT-PCR: quantitative reverse transcription PCR; NC shRNA: negative control short hairpin RNA; Runx2: runt-related transcription factor 2; α-SMA: alpha smooth muscle actin. *N* = 3. At least two independent experiments were performed. *p < 0.05 vs. the NC shRNA group.

**Figure 3.**
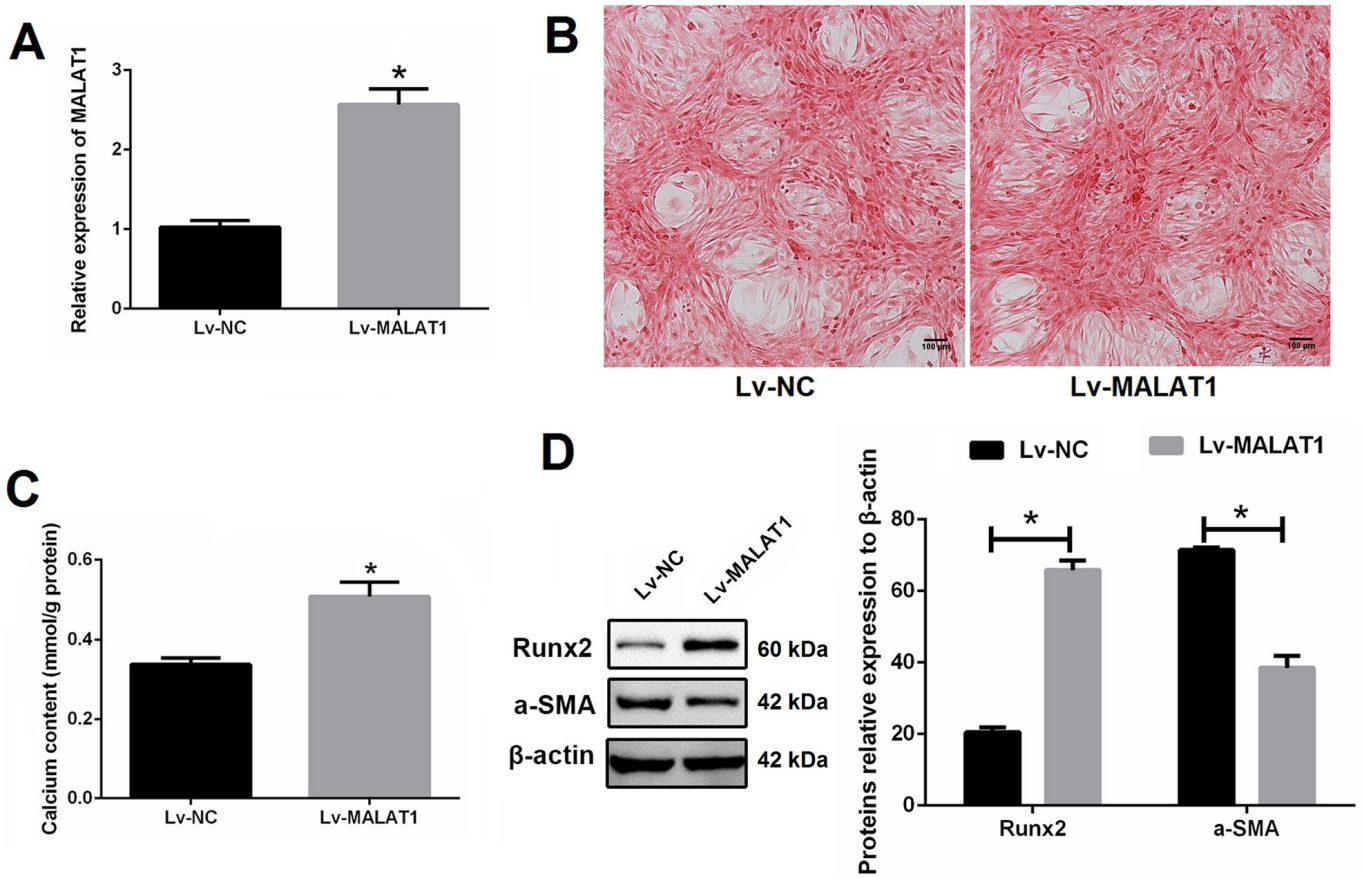
Up-regulation of MALAT1 promoted VSMCs calcification. (A) qRT-PCR showed that the expression of MALAT1 was significantly up-regulated after Lv-MALAT1 transfection; (B) Alizarin red S staining indicated more mineralized nodules in the Lv-MALAT1 group; (C) calcium assay demonstrated higher calcium content in the Lv-MALAT1 group; (D) Western blot demonstrated higher expression of Runx2 and lower expression of α-SMA in the Lv-MALAT1 group. MALAT1: metastasis-associated lung adenocarcinoma transcript 1; VSMCs: vascular smooth muscle cells; qRT-PCR: quantitative reverse transcription PCR; Lv-NC: negative control lentivirus; Lv-MALAT1: MALAT1 lentivirus; Runx2: runt-related transcription factor 2; α-SMA: alpha smooth muscle actin. *N* = 3. At least two independent experiments were performed. *p < 0.05 vs. the Lv-NC group.

To explore the role of MALAT1 in VSMCs calcification, lentiviral shRNA of MALAT1 was constructed. As expected, the expression of MALAT1 was strikingly decreased after MALAT1 shRNA transfection as compared with negative control shRNA (NC shRNA) transfection (Figure 2(A)). MALAT1 knockdown greatly suppressed VSMCs calcification, as indicated by less mineralized nodules (Figures 2(B) and S2), lower calcium content (Figure 2(C)), lower expression of Runx2 and higher expression of α-SMA (Figure 2(D)). On the contrary, over-expression of MALAT1 using a lentiviral vector (Lv- MALAT1) (Figure 3(A)) promoted VSMCs calcification (Figures 3(B–D) and S3).

### MALAT1 directly sponges miR-30c, up-regulation of which suppressed VSMCs calcification

**Figure 4.**
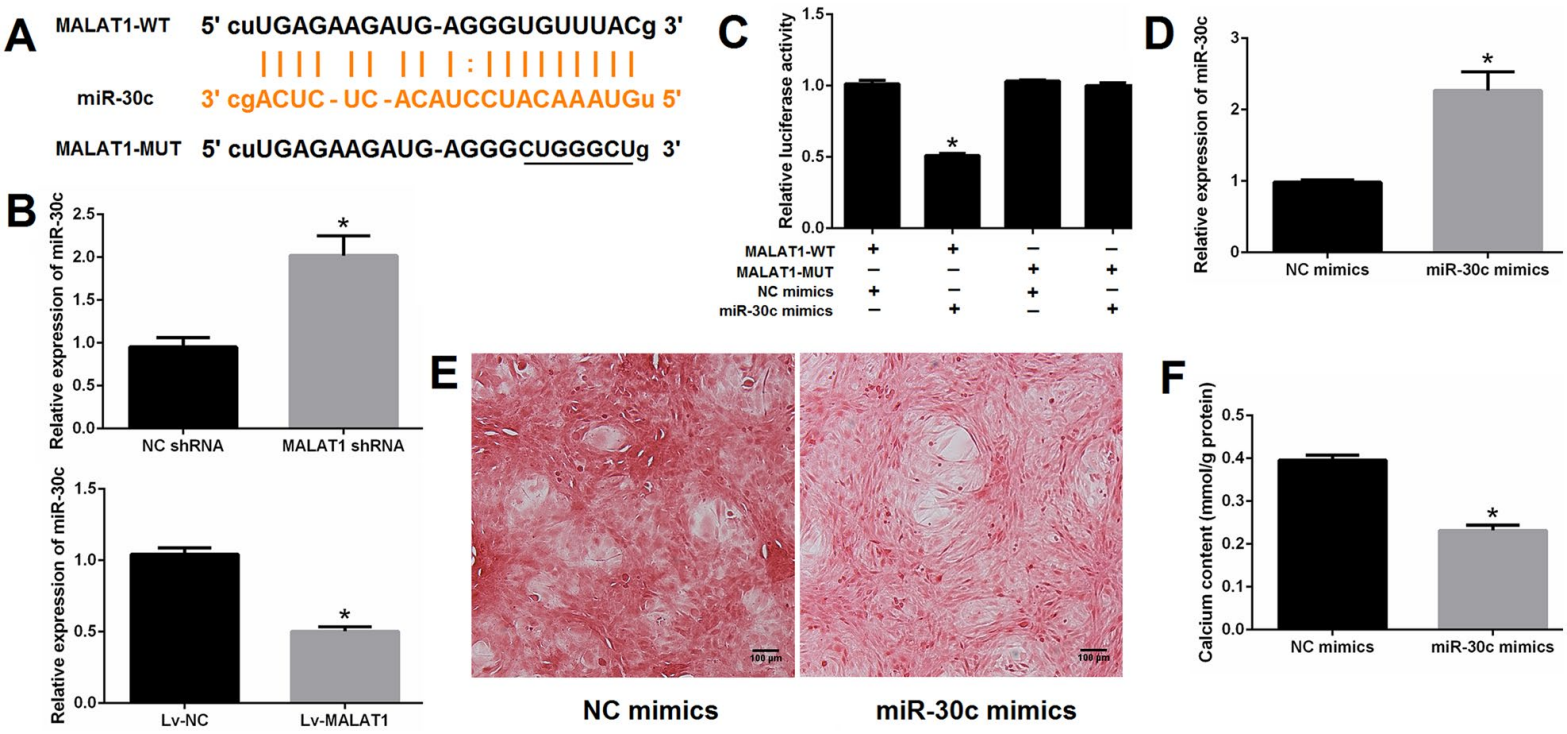
MALAT1 directly sponged miR-30c. (A) putative binding sites of MALAT1 with miR-30c as predicted by the ENCORI platform; (B) qRT-PCR showed that miR-30c expression was increased after MALAT1 knockdown and decreased after MALAT1 up-regulation; (C) Dual-luciferase reporter assay indicated that miR-30c mimics could significantly reduce the luciferase activity of cells transfected with MALAT1-WT; (D) qRT-PCR showed that miR-30c expression was increased after miR-30c mimics transfection; (E and F) up-regulation of miR-30c with mimics promoted VSMCs calcification as indicated by Alizarin red S staining and calcium assay. MALAT1: metastasis-associated lung adenocarcinoma transcript 1; qRT-PCR: quantitative reverse transcription PCR; NC shRNA: negative control short hairpin RNA; Lv-NC: negative control lentivirus; Lv-MALAT1: MALAT1 lentivirus. *N* = 3. At least two independent experiments were performed. *p < 0.05 vs. NC groups or cells cotransfected with MALAT1-WT and NC mimics.

To verify the relationship between MALAT1 and miR-30c, the binding site of MALAT1 and miR-30c was predicted using the ENCORI platform (https://starbase.sysu.edu.cn) (Figure 4(A)). qRT-PCR results showed that the expression of miR-30c was strongly increased after MALAT1 knockdown but decreased after MALAT1 up-regulation, implying the suppressive effect of MALAT1 on miR-30c (Figure 4(B)). Dual-luciferase report assay further demonstrated that miR-30c mimics, with no effect on cells transfected with MALAT1-MUT reporter plasmid, could markedly reduce the luciferase activity of cells transfected with MALAT1-WT reporter plasmid (Figure 4(C)). These results suggested a direct interaction between MALAT1 and miR-30c.

Next, to explore the role of miR-30c in VSMCs calcification, miRNA mimics was transfected. As expected, the expression of miR-30c was significantly increased after miR-30c mimics transfection as compared with NC mimics transfection (Figure 4(D)). Up-regulation of miR-30c greatly suppressed VSMCs calcification, as indicated by Alizarin red S staining (Figures 4(E) and S4) and calcium assay (Figure 4(F)). Taken together, our results demonstrated that MALAT1 could directly sponge miR-30c, up-regulation of which suppressed VSMCs calcification.

### Runx2 is a direct target of miR-30c

**Figure 5.**
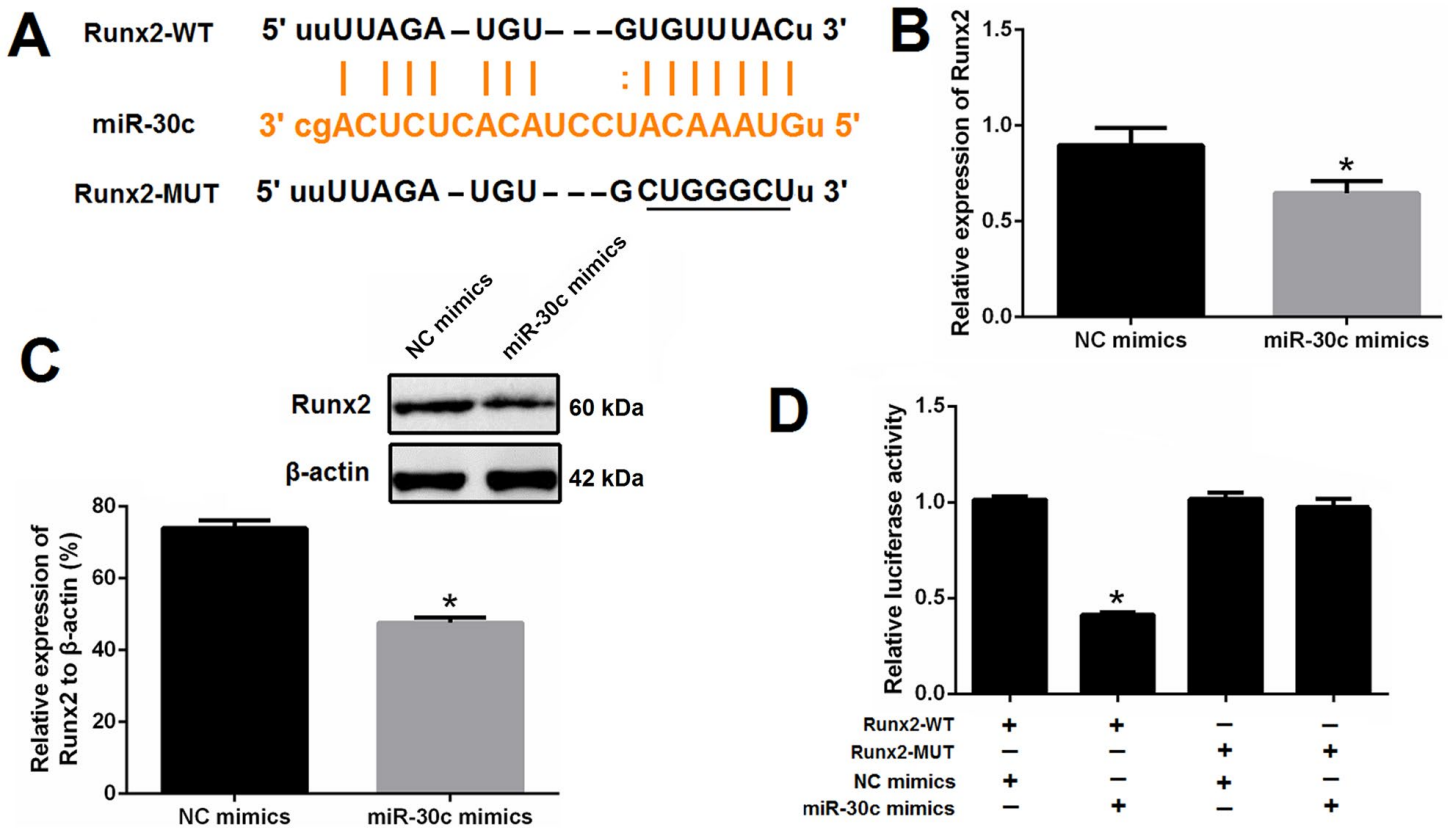
Runx2 is a direct target of miR-30c. (A) miR-30c putative target site in the 3’-untranslated region of Runx2 as predicted by the ENCORI platform; (B and C) qRT-PCR and western blot showed that Runx2 expression was decreased after miR-30c mimics transfection; (D) dual-luciferase reporter assay indicated that miR-30c mimics could significantly reduce the luciferase activity of cells transfected with Runx2-WT. MALAT1: metastasis-associated lung adenocarcinoma transcript 1; Runx2: runt-related transcription factor 2; qRT-PCR: quantitative reverse transcription PCR. *N* = 3. At least two independent experiments were performed. *p < 0.05 vs. NC groups or cells cotransfected with Runx2-WT and NC mimics.

To confirm whether Runx2 is a direct target of miR-30c, the ENCORI platform was used to obtain the binding sites of miR-30c and Runx2 (Figure 5(A)). Next, qRT-PCR and western blot results revealed that the expression of Runx2 was significantly decreased after miR-30c mimics transfection as compared with NC mimics transfection (Figure 5(B,C)). Dual-luciferase report assay further demonstrated that miR-30c mimics could prominently reduce the luciferase activity of cells transfected with Runx2-WT reporter plasmid; however, this effect was not observed in cells transfected with Runx2-MUT reporter plasmid (Figure 5(D)). These results suggested Runx2 is a direct target of miR-30c.

### The effect of MALAT1 on VSMCs calcification was at least partially through regulating the miR-30c/Runx2 axis

**Figure 6.**
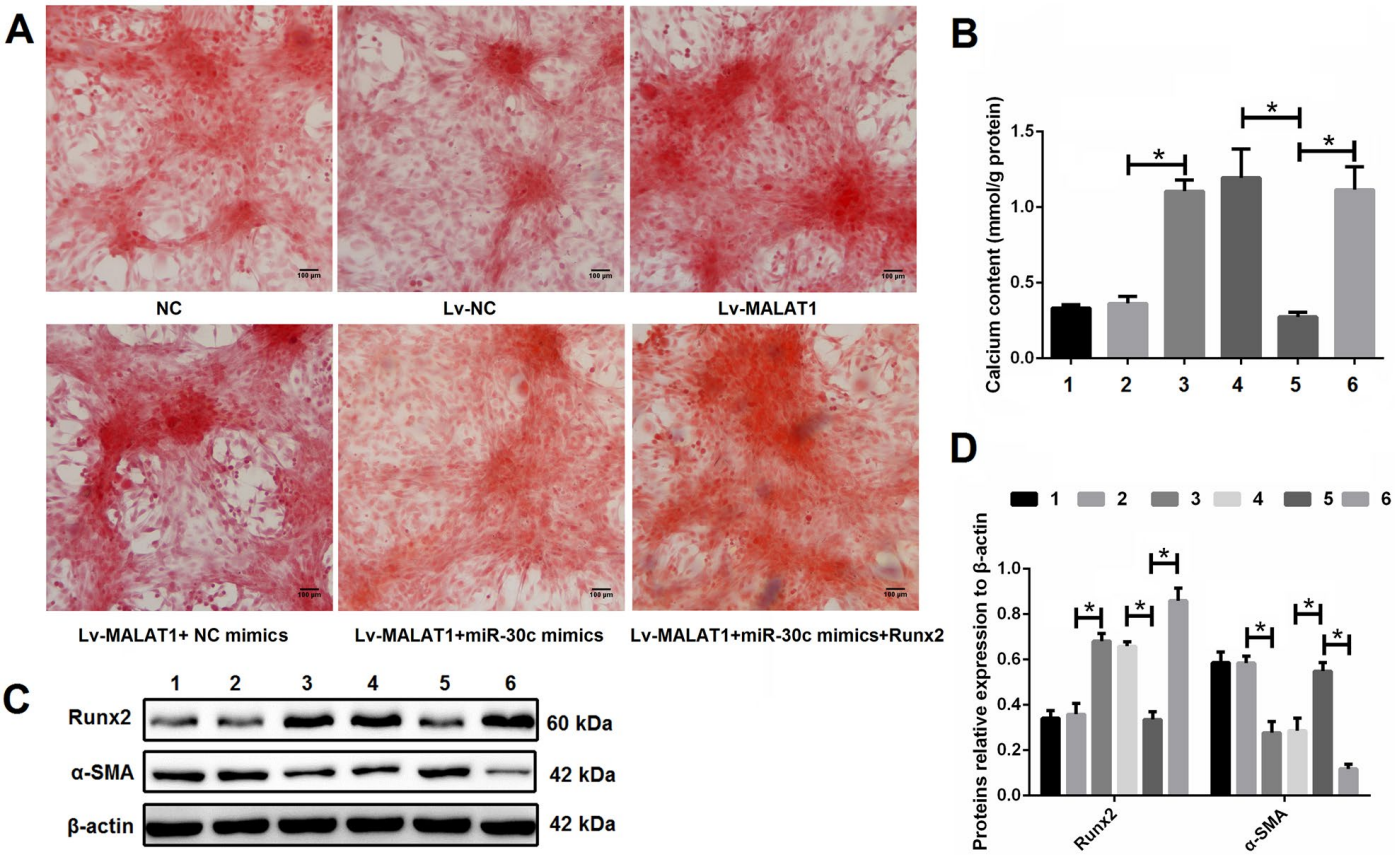
The effect of MALAT1 on VSMCs calcification was at least partially through regulating the miR-30c/Runx2 axis. (A) Representative Alizarin red S staining pictures; (B) calcium assay showed that the Lv-MALAT1 vector induced higher calcium content, which was decreased after adding miR-30c mimics but increased again after adding Runx2 over-expression vector; (C and D) Western blot results showed that Lv-MALAT1 vector increased Runx2 but decreased α-SMA expression, this effect was reversed after adding miR-30c mimics but reversed again after adding Runx2 over-expression vector. 1 represent the NC group, 2 represent the Lv-NC group, 3 represent the Lv-MALAT1 group, 4 represent the Lv-MALAT1 + NC mimic group, 5 represent the Lv-MALAT1 + miR-30c group and 6 represent the Lv-MALAT1+ miR-30c mimic + Runx2 group. NC: negative control; Lv-NC: negative control lentivirus; Lv-MALAT1: MALAT1 lentivirus; Runx2: runt-related transcription factor 2; α-SMA: alpha smooth muscle actin. *N* = 3. At least two independent experiments were performed. *p < 0.05.

To elucidate the regulatory relationship between MALAT1, miR-30c and Runx2, calcified VSMCs were treated with DMEM medium (NC group), empty vector (Lv-NC group), Lv-MALAT1 vector (Lv-MALAT1 group), Lv-MALAT1 vector + NC mimic (Lv-MALAT1 + NC mimic group), Lv-MALAT1 vector + miR-30c mimic (Lv-MALAT1 + miR-30c group) or Lv-MALAT1 vector + miR-30c mimic + Runx2 over-expression vector (Lv-MALAT1+ miR-30c mimic + Runx2 group). Our results showed that over-expression of MALAT1 with Lv-MALAT1 vector significantly promoted VSMCs calcification (Figures 6(A,B) and S5), increased Runx2 but decreased α-SMA expression (Figure 6(C,D)); unsurprisingly, upregulation of miR-30c with miR-30c mimic reversed this effect, which was reversed again by upregulation of Runx2 with over-expression vector (Figures 6(A–D) and S5), indicating that the effect of MALAT1 on VSMCs calcification was at least partially through regulating the miR-30c/Runx2 axis.

## Discussion

VSMCs are the predominant cells in the vessel media and frequently used to study medial VC [18]. In this research, we induced *in vitro* VC model in VSMCs with high-phosphate medium and our results demonstrated that MALAT1 expression was increased, while miR-30c expression was decreased in calcified VSMCs. Mechanically, MALAT1 promoted VSMCs calcification, which was at least partially through regulating the miR-30c/Runx2 axis.

MALAT1 was initially identified in non-small cell lung cancer and subsequently reported to be highly expressed in numerous cancers including but not limited to bladder cancer, gastric cancer, hepatocellular carcinoma, breast cancer, colorectal cancer, ovarian cancer, prostate cancer and renal cell carcinoma [19]. MALAT1 also participates in the pathogenesis of several VC-related diseases. For instance, Xiao et al. demonstrated that MALAT1 was highly expressed in calcified aortic valves tissues and promoted osteogenic differentiation of VICs by sponging miR-204 [13]. Sohrabifar et al. revealed that MALAT1 was significantly up-regulated in patients with coronary artery disease (CAD) and DM; besides, MALAT1 had the highest diagnostic power for discrimination of CAD patients from controls. Authors concluded that MALAT1 participates in the pathogenesis of CAD and DM and may be applied as a potential diagnostic marker for CAD [20]. In a review article of Abdulle et al. authors focused on the correlation between MALAT1 and diabetes-related complications (including cerebral ischemic reperfusion injury, diabetic retinopathy, diabetic cataract, atherosclerosis, diabetic cataract, diabetic cardiomyopathy, nonalcoholic steatohepatitis, diabetic gastropathy, diabetic kidney disease and gestational DM) and highlighted that MALAT1 is a promising diagnostic and therapeutic target for these conditions [21]. In another study performed by Yu et al. authors demonstrated MALAT1 is a stiffness-sensitive lncRNA that regulates stiffness-dependent VSMC proliferation and migration *in vitro* and *in vivo* [22]. In our study, we found the expression of MALAT1 was up-regulated in calcified VSMCs. Knockdown of MALAT1 with shRNA suppressed VSMCs calcification; on the contrary, up-regulation of MALAT1 with over-expression plasmid promoted VSMCs calcification. Collectively, these evidence verified the important roles of MALAT1 in VC, suggesting that MALAT1 may serve as a therapeutic target for VC-related diseases.

According to the competitive endogenous RNA (ceRNA) hypothesis, lncRNAs can modulate the expression of downstream genes by competitively binding to miRNAs. MALAT1 may function as a ceRNA to participate in the process of VC. To further delineate the molecular mechanism by which MALAT1 modulates VC, miR-30c was focused because miR-30c, as a well known target of MALAT1 [15–17], was found to be associated with the pathogenesis of VC [14]. In a previous study performed by Joshua et al. authors found bone morphogenetic protein-2 decreased miR-30b and miR-30c expression in human coronary artery SMCs, leading to vascular calcification [23]. Ciceri et al. demonstrated that the anti-calcific effect of iron citrate in VSMCs was relevant with the increase of miR-30c [24]. In the present study, the expression of miR-30c was decreased in calcified VSMCs and up-regulation of miR-30c with miRNA mimics suppressed VSMCs calcification, which was in accordance with previous studies [23,24]. In addition, MALAT1 sponges miR-30c in VSMCs was confirmed by our results. Firstly, we predicted that MALAT1 contains binding sites for miR-30c using the ENCORI platform and confirmed their direct binding relationship using dual-luciferase report assay; Secondly, we found that MALAT1 was inversely related to miR-30c in VSMCs; Thirdly, the effect of MALAT1 over-expression on VSMCs calcification was reversed by up-regulation of miR-30c.

Runx2, a well known target of miR-30c [16,23,25], is essential for osteoblastic differentiation and VSMCs calcification [26–28]. In the present study, we found Runx2 was up-regulated in calcified VSMCs and upregulation of Runx2 could reverse the effects of miR-30c increase on VSMCs calcification. Taken together, our results verified that MALAT1 sponged miR-30c to up-regulate the expression of Runx2 and thus promoted VSMCs calcification.

## Conclusions

MALAT1 over-expression promoted VSMCs calcification, which was at least partially through mediating the miR-30c/Runx2 axis. MALAT1 may serve as a potential therapeutic target for VC-related diseases.

## Supplementary Material

Supplemental Material
