# Supplementary material for: Long non-coding RNA MALAT1 sponges miR-30c to promote the calcification of human vascular smooth muscle cells by regulating Runx2
Source: Ren Fail. 2023 Apr 26;45(1):2204953. doi: 10.1080/0886022X.2023.2204953 (PMC10134953; doi:10.1080/0886022X.2023.2204953)
Supplement: Supplemental Material [file IRNF_A_2204953_SM9029.pdf]

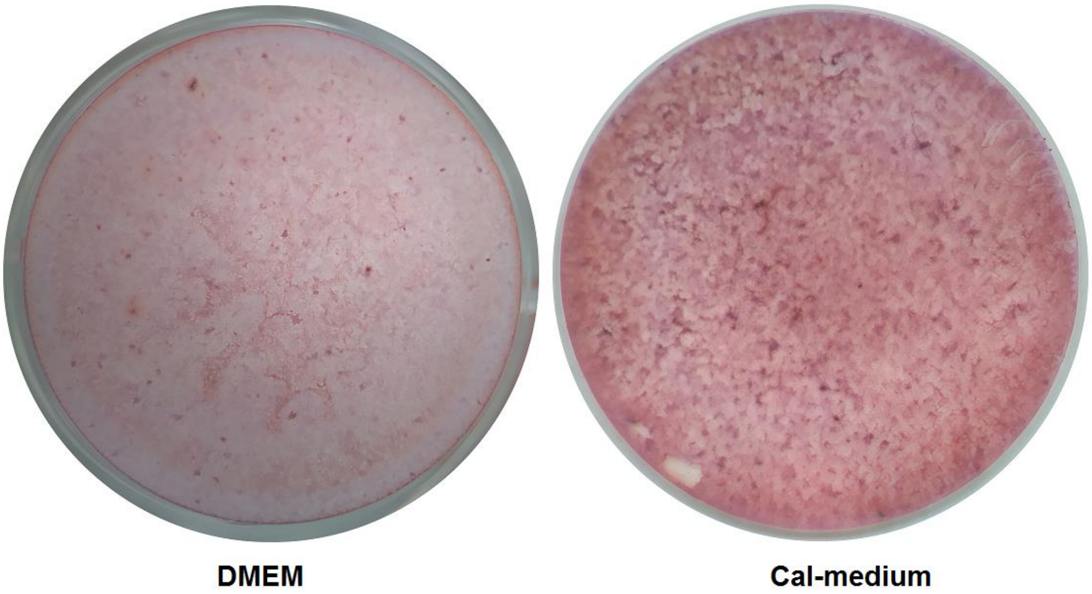

Figure S1. The whole pictures of alizarin red staining of the DMEM group and the Cal-medium group

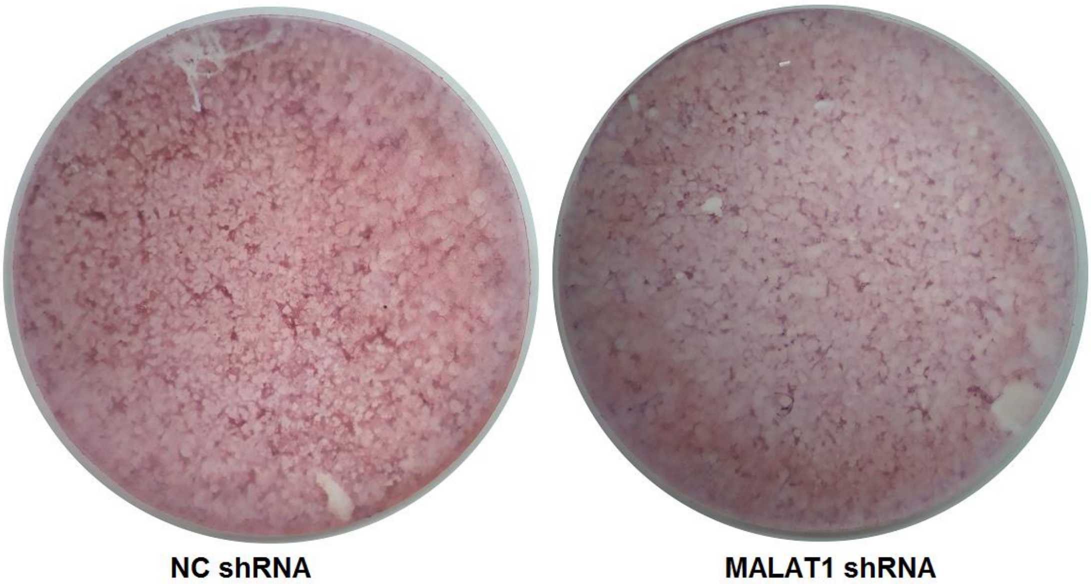

Figure S2. The whole pictures of alizarin red staining of the NC shRNA group and the MALAT1 shRNA group

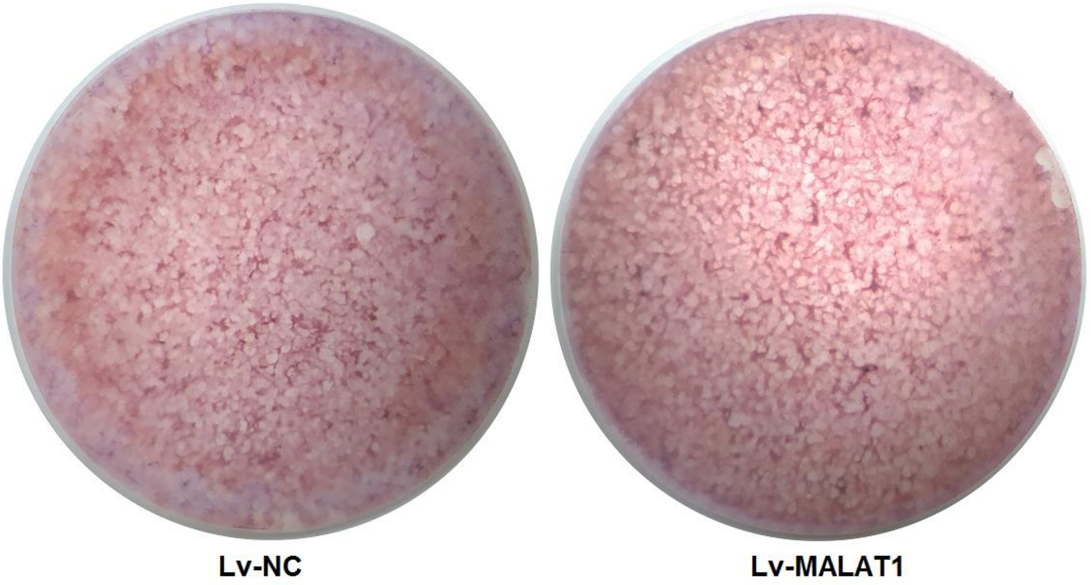

Figure S3. The whole pictures of alizarin red staining of the Lv-NC group and the Lv-MALAT1 group

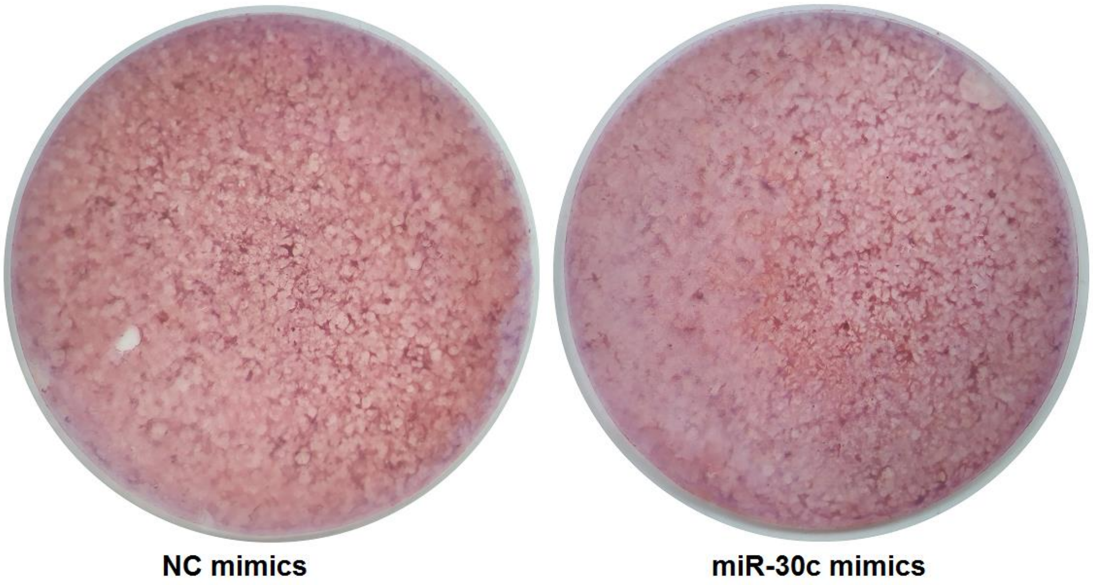

Figure S4. The whole pictures of alizarin red staining of the NC mimics group and the miR-30c mimics group

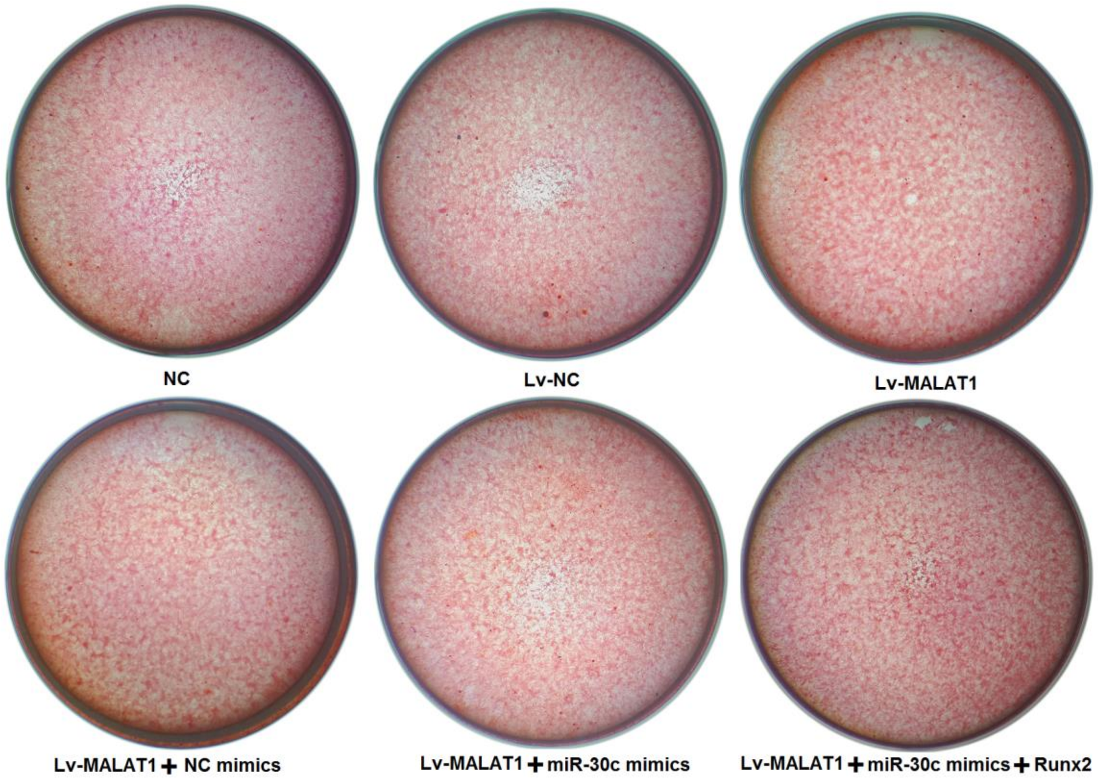

Figure S5. The whole pictures of alizarin red staining
